# Measurement of Axial Rigidity and Postural Instability Using Wearable Sensors

**DOI:** 10.3390/s18020495

**Published:** 2018-02-07

**Authors:** Dung Phan, Malcolm Horne, Pubudu N. Pathirana, Parisa Farzanehfar

**Affiliations:** 1 School of Engineering, Deakin University, Waurn Ponds, VIC 3216, Australia; thimydun@deakin.edu.au; 2 Florey Institute of Neuroscience and Mental Health, Parkville, VIC 3052, Australia; malcolm.horne@florey.edu.au (M.H.); parisa.farzanehfar@florey.edu.au (P.F.)

**Keywords:** biomedical signal processing, Parkinson’s disease, bradykinesia, rigidity, flexibility, damping ratio, principal component analysis

## Abstract

Axial Bradykinesia is an important feature of advanced Parkinson’s disease (PD). The purpose of this study is to quantify axial bradykinesia using wearable sensors with the long-term aim of quantifying these movements, while the subject performs routine domestic activities. We measured back movements during common daily activities such as pouring, pointing, walking straight and walking around a chair with a test system engaging a minimal number of Inertial Measurement (IM) based wearable sensors. Participants included controls and PD patients whose rotation and flexion of the back was captured by the time delay between motion signals from sensors attached to the upper and lower back. PD subjects could be distinguished from controls using only two sensors. These findings suggest that a small number of sensors and similar analyses could distinguish between variations in bradykinesia in subjects with measurements performed outside of the laboratory. The subjects could engage in routine activities leading to progressive assessments of therapeutic outcomes.

## 1. Introduction

Bradykinesia is the key motor symptom of Parkinsons Disease (PD) [1]. However, distinctions are made between subjects with PD with limb bradykinesia and/or tremor (so called tremor dominant PD) and subjects with postural instability, axial rigidity and gait disorder predominate (PIGD) [1] because of differing prognosis. Postural instability may result from several different mechanisms, but the term is usually reserved for a disturbance of posture as measured by the pull test. It is manifested as impaired standing, walking or turning and lead to falls and a fear of falling [2,3]. Postural body sway is reduced in PD. While this may be to compensate for the instability that results when the centre of mass is displaced, loss of postural reflexes is well recognised in PD [2]. Axial rigidity may also contribute to reduced body sway. In PD, rigidity is usually used to refer to increased distal muscle tone and testing of axial rigidity is difficult at the bedside.

The development of wearable devices has made the measurement of distal bradykinesia possible [3,4,5]. However, there is also a need for ambulatory measurement of axial rigidity and postural instability in an ambulatory setting [6]. Steps have been made in assessing postural instability [7,8], but a system that is to be worn for some days in routine care will require that instrumentation be kept to a minimum [6].

The pull test is widely used as a method of assessing postural instability. In this exercise, the patient is pulled backwards by the shoulders with sufficient force to dislodge the centre of gravity backwards and cause corrective movements of the trunk and a backwards step to recover postural stability [7]. People with PD take more shuffling steps to regain stability, and indeed may fail to recover stability and would fall if not supported by the testing clinician. Axial rigidity and poor trunk coordination in PD result in a reduced stability margin [9] and increased vulnerability to backward falls [8]. The Hoehn and Yahr scale (H&Y) is a clinical rating scale, which is a widely used and accepted staging system. It defines the severity of PD dysfunction including disability and impairment by stage I through V [10]. The severity levels are rated from unilateral (I) to bilateral disease (II) without impairment of balance, to the presence of postural instability (III), loss of physical independence (IV), and wheelchair bound or bedridden (V). It has been used in this study to correlate with the analysis.

This paper describes a preliminary step to ask whether two sensors were sufficient to extract information regarding instability and rigidity from people who had had bilateral PD that affected postural stability, which is a Hoehn and Yahr classification of III (H&Y III) or more. This is meant as a prelude to further studies examining the threshold of this approach to more minor impairments of posture or axial rigidity and whether variation of the course of a dose of levodopa can be detected. In this study, subjects wore sensors that measured angular rate (BioKin^TM^) and were located on each shoulder and each posterior iliac spine. The findings were that people with PD (of severity of H&Y III or greater) could be distinguished from aged matched controls by a delay between angular rate measured from the shoulder and hip on the same side and by features in the frequency domain extracted by principal component analysis. The main insights from this study are that the axial rigidity and postural instability in the pointing, pouring, walking in a straight line and walking around a chair tests can be measured using two sensors based on the feature that uses the latency between the hip and shoulder sensors and their frequency content. This provides the preliminary data to justify further studies comparing subjects with a wide range of axial involvement with PD and with motor fluctuations.

## 2. Experimental Setup

**Figure 1:**
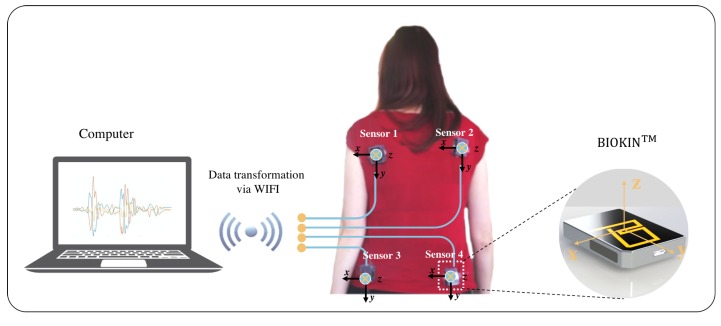
Experiment setup: Four BioKin${}^{\mathrm{TM}}$ sensors are attached on the back, connected to computer via wifi.

For this study, movements that mimicked regular day-to-day activities but required back motion were standardized. Movements that entailed pouring, pointing, walking, and walking around a chair were performed according to a protocol to assess whether the flexibility of the human back could be measured. As described above, the tasks were selected to capture the presence of the automatic movements of the lumber and thoracic spine required in carrying out these goals’ directed tasks. The aim was to capture the rotation and flexion of the shoulders on the hip with the assumption, which in the presence of axial bradykinesia and torsal rigidity would be altered. BioKin^TM^ uses the chip (MPU-9150) from the manufacturer InvenSense, Inc. San Jose, CA, USA, which is optimized to reduce settling effects and sensor drift by elimination of board-level cross-axis alignment errors between accelerometer, gyroscope and compass [11]. Four of these devices were used in this study to capture angular rate and acceleration data. This study was approved by the Human Research and Ethics Committee, St. Vincent’s Hospital Melbourne, Australia and written consent forms were signed by participants prior to participation. The ethics number is HREC A 152/09. In this research, there were about eight control subjects and 15 people with PD of severity H&Y III. Controls and people with PD were of similar age (68.8 ± 6.9 years). Participants wore four sensors on the back with two sensors on the upper left/right and two sensors on the lower left/right, as shown in Figure 1. For each activity, patients and controls are outlined in Table 1.

**Figure 2:**
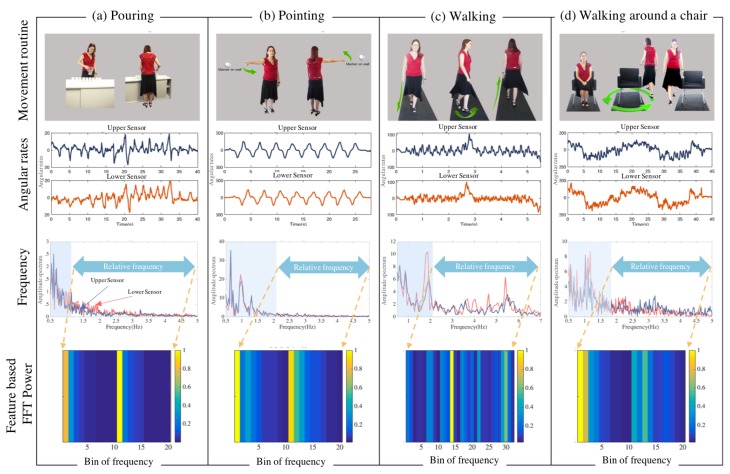
Experimental setup and analysis. Figure (**a**–**d**) demonstrates movement routine for pouring, pointing, walking and walking around a chair; the second row depicts angular rates in time domain from sensors at the upper and lower; the third row indicates frequency domain (FFT) representation for each of the movement routine. Here, upper sensor (red) and lower sensor (blue), and the dominant frequency band is indicated in a shaded area; the fourth row shows examples of power spectral density of the relevant band forming the feature vectors.

In the pouring test, nine cups were aligned in a row at roughly hip height, and participants were required to pour water from a jug sequentially into each cup, as demonstrated in Figure 2a. This test requires both axial rotation and distal dexterity. It also allows bradykinesia and rigidity to be highlighted by shifting attentional focus toward the distal task and away from the automatic proximal movements required as a platform for the distal movements [12]. Thus, impaired control of either axial movement and distal limb movement [13] can be observed. It may also be possible to calculate axial rigidity from these movements. While not the focus of this study, upper limb tremor may be evident in the distal extremity involvement of patients performing the pouring test [14].

The pointing test also requires truncal rotation. In this exercise, two markers were placed on opposite walls. Subjects are required to stand, keeping the feet stationary and using the same hand point from marker 1 to marker 2 while attempting to rotate the shoulders through 180° and back. This task was executed approximately ten times, as shown in Figure 2b without turning the feet. The pouring and pointing tasks were chosen because they require attentional gaze to be on the activities of the upper limbs but automatic adjustment of the trunk through rotation and flexion required as a platform for the distal movements [12]. The focus in both tasks is to measure axial bradykinesia and possibly to calculate axial rigidity. While not the main focus of this study, impaired control of either distal limb movement [13] and tremor can also be observed [14].

In the walking test, the patient is required to walk forward in a straight line, turn 180° and walk in the opposite direction, approximately five times, as indicated in Figure 2c. As bradykinesia is typical of Parkinsonian gait, slow walking velocity, small stride length and shuffling steps are expected to be observed during the walking phase of this exercise [15].

The walking around a chair test begins with the subject seated in a chair. They are then required to stand up and walk around the chair and finish the routine by sitting down, as depicted in Figure 2d. It is expected that axial rigidity and postural instability will be made apparent during the standing phase of this exercise.

Normal walking requires motion of the pelvis, trunk and shoulders and turning requires considerable axial rotation. It is expected that, in both tasks, PD subjects will have reduced back motion with smaller step size resulting in a greater number of steps [14,15,16]. It is expected that motion of the back will be altered by PD in both tasks with difficulty in turning. Initiating turns are particularly difficult for PD patients due to reduced cortical activation as a result of basal ganglia dysfunction [17]. Axial rigidity is expected to have a role causing difficulties during the turning phase of the exercise.

Angular rate signals were recorded by the four sensors and transmitted via wireless means for signal processing. Typical temporal variation of the signals of the four tests are depicted in Figure 2.

## 3. Activity Analysis

Angular rate data from four sensors were captured during the routine movements. A low-pass filter with cutoff frequency of 10 Hz was engaged in preprocessing the angular rate signals to exclude higher frequency noise.

In this study, we considered three independent analyses to observe varying aspects with minimal number of sensor utilisation in human flexibility capture. Firstly, we looked at the pointing test data using a second order description of the underling rotational movements. This is indeed based on describing human back rigidity in terms of system’s theoretic characterisations in the form of resonant frequency and damping coefficients.

**Figure 3:**
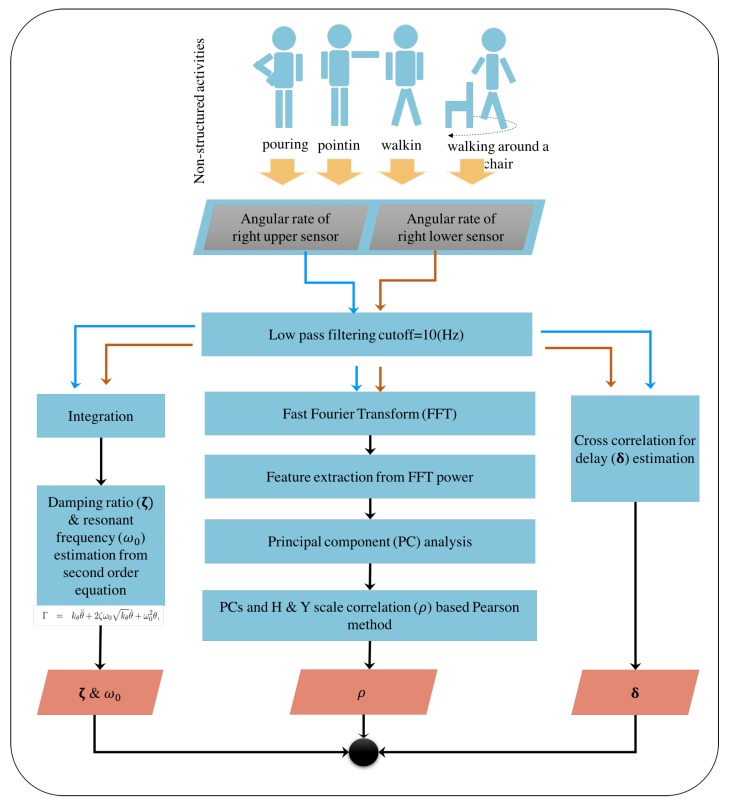
Unstructured activity analysis for Parkinsonian features: Blue arrows represented the sequence of the upper sensor data processing; red arrows represented the sequence of the lower sensor data processing; and black arrows represented the common sequence of both upper and lower sensor processing.

The second and third approaches were based on the analysis of data from two sensors from the upper and lower back based on delay time and feature described in frequency domain, respectively. A graphical description highlighting these distinctive approaches is illustrated in Figure 3.

### 3.1. Resonant Frequency and Damping Coefficient with a Single Sensor

Axial rotation can be considered as rigid body rotational movement described as

(1) $$\begin{array}{ccc} \Gamma & = & k_{\theta }\ddot{\theta }+2\zeta \omega_{0}\sqrt{k_{\theta }}\dot{\theta }+\omega_{0}^{2}\theta , \end{array}$$

where $\Gamma ,k_{\theta }$, θ, ζ, and $\omega_{0}$ are the applied torque, Young’s rotational modulus, the angle of rotation, damping coefficient and the resonant frequency, respectively. Our assumption is that, when performing the axial rotation required for the movements in these studies relevant to daily activities, approximately similar torques are exerted and therefore the effects of bradykinesia are able to be observed in angular accelerations. This is a reasonable assumption for normal subjects of all ages [18] and we assume here that it will be similar in PD. We inadvertently acquire the rigidity information, which is intrinsically embedded in flexibility coefficients (i.e., $k_{\theta }$). As given in [19], the derivative operator can be given as a linear combination of the Fourier coefficients, as seen in Equation (2):

(2) $$\begin{array}{cc} \ddot{\theta } & =\frac{d\dot{\theta }\left(t\right)}{dt}\\ & =\sum_{0<k<N/2}\frac{2\pi i}{L}k\left(F_{k}e^{+\frac{2\pi i}{L}kt}-F_{N-k}e^{-\frac{2\pi i}{L}kt}\right)-\\ & -\frac{\pi }{L}NF_{N/2}\sin\left(\frac{\pi }{L}Nt\right),\;\mathrm{where}\;F_{k}=\frac{1}{L}\int_{0}^{L}e^{-\frac{2\pi i}{L}kt}\dot{\theta }\left(t\right)dt, \end{array}$$

where θ(t) is the angle of rotation at time *t*; $F_{k}$ is the Fourier coefficients at *k*th time frame and *N* is the time frame number.

Therefore, the spectral coefficients of the measured angular velocities directly correspond to the angular accelerations and inversely linked to the Young’s rotational modulus ($k_{\theta }$). Hence, we look at the spectral components of the relevant (dominant) bands of the measured angular velocity in distinguishing bradykinesia as feature vectors for deducing principal components in the classification of PD patients.

**Figure 4:**
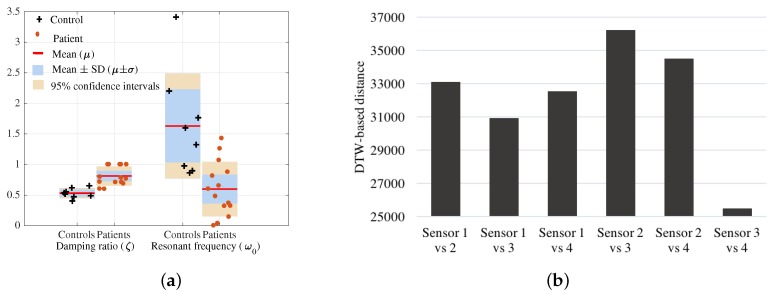
(**a**) Damping ratio (ζ) and resonant frequency ($\omega_{0}$) deduced from the pointing test; (**b**) distance between angular rate of each pair of sensors using dynamic time warping (DTW).

In the pointing test, we estimated the damping ratio (ζ) and resonant frequency ($\omega_{0}$) for each subject from the second order differential system of motion. Figure 4a shows that the ζ values of controls (0.4≤ζ≤0.68) are less in comparison to the patients (0.65≤ζ≤1) indicating axial damping due to Parkinsonism. The resonant frequency values of patients are smaller in comparison to controls as dominant movements or the activity is conducted at a slower rate due to bradykinesia.

### 3.2. Truncal Flexibility Analysis with Two Sensors

The rigidity of human body can be observed through the upper back (Sensor 1 and Sensor 2) and lower back (Sensor 3 and Sensor 4) during the rotation. We first observe *how different* the data is from the four sensors. Here, we resort to the *distance* between signals from the upper and lower sensors using dynamic time warping (DTW) [20]. Pairs of sensors that gave the largest signals distance/difference based on DTW was selected for the analysis. A brief description on DTW is provided in Section 3.2.1.

Firstly, (Section 3.2.2), with two sensors, we extracted the delay between signals from a pair of sensors based on the cross validation method for four separated movement routines to show the differences between controls and patients groups.

Secondly, (Section 3.2.3), in the primary analysis undertaken in this research, the frequency domain features extracted were optimised against feature parameters using the Silhouette coefficient. Then, principal component analysis (PCA) was applied to extract significant features. These techniques have been applied for all the four tests independently to describe features in each movement routine. The extracted PCA features from each movement routine were fused using PCA to generate new features expressing the combination of all tests.

#### 3.2.1. Minimising the Number of Sensors

The DTW measures the similarity between two time signals based on warping paths. The optimal match between two sequences allowing shifting, stretching and compression of sections of the sequence is obtained. Therefore, DTW based distance was utilized in this study to obtain the combination of signals from sensors that contained the most information (large difference/distance) for the subsequent analysis. In order to observe the truncal rigidity, we consider vertical axis rotation characteristics of the human back that is captured by the angular rate around the *x*-axis. Therefore, the *distance* between signals (angular rates) from distinct sensors were obtained by means of DTW. The DTW distance between signals from each pair of sensors was calculated for each routine movement. Then, the average value of the DTW distances for each movement activity is used to quantify the signal deviation from each sensor pair. In Figure 4, the similarity scale is the average DTW distance between signals from each pair of sensors for each activity (pointing, pouring, walking and walking around a chair). The study observed six pairs of sensors (Sensor 1 vs. 2, Sensor 1 vs. 3, Sensor 1 vs. 4, Sensor 2 vs. 3, Sensor 2 vs. 4, Sensor 3 vs. 4) that are depicted in Figure 4b. The two sensor pairs, Sensor 2 vs. 4 and Sensor 2 vs. 3 produced the largest *distance* between the upper and lower back and hence were selected as the dominant sources for back flexibility information in capturing axial bradykinesia for this study.

#### 3.2.2. Flexibility Analysis with Time Delay Information

We evaluated back flexibility as well as spinal flexibility, which are related to functional limitations. The slower movements with less displacement, typical of Parkinson’s patients, were attributed to the lower damping ratio captured by the sensors. The rotation of the human back is captured by the *x*-axis Gyroscopic reading. In order to quantify back flexibility, we obtained the delay between two signals from two sensors attached at the upper and the lower back. This directly corresponded with the damping effects of the subject movements. The delay between two angular velocity signals $S_{T}$ and $S_{B}$ is calculated based on cross correlation function as in Equations (3) and (4):

(3) $$R_{S_{T},S_{B}}\left(m\right)=\left\{\begin{array}{c} \sum_{n=0}^{N-m-1}S_{1,n+m}S_{2,n}^{*},m\geq 0,\\ R_{S_{B},S_{T}}\left(-m\right),m<0, \end{array}\right.$$

(4) $$xcross\left(m\right)=R_{S_{T},S_{B}}\left(m-N\right),m=1,2,\ldots ,2N-1.$$

Then, the normalised cross-correlation between each pair of signals is calculated. The captured delay δ is given by the negative of the lag for which the normalised cross-correlation has the largest absolute value. Mean μ and the standard deviation σ of delay are calculated using Equation (5) to investigate the difference between control and patient groups. The equations are as below:

(5) $$\mu =\frac{\delta_{1}+\delta_{2}+\ldots +\delta_{m}}{m},\sigma =\sqrt{\frac{\sum_{i=1}^{m}\left(\delta_{i}-\mu \right)}{m-1}}.$$

**Figure 5:**
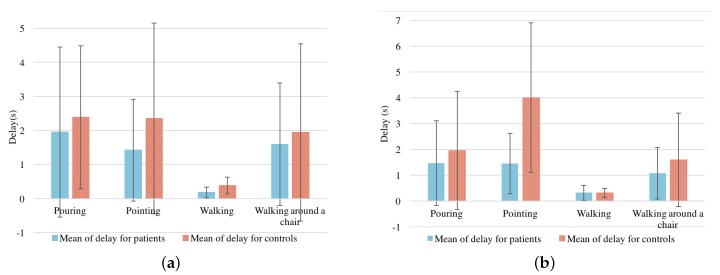
Mean (μ) ± standard variation (σ) of the delay time between sensors: (**a**) Mean (μ) ± standard variation (σ) of the delay time between Sensor 2 and Sensor 3, (**b**) Mean (μ) ± standard variation (σ) of the delay time between Sensor 2 and Sensor 4.

Means and standard deviations of time delays were calculated and depicted in Figure 5a for Sensor 2 vs. 3, and Figure 5b for Sensor 2 vs. 4. This minimal delay between the upper and lower of the backs of PD patients while walking is due to slow movements and small steps. The delays are longer for controls than for patients in each test, except the walking test, and are significantly different in the pointing test. We propose that increased flexibility and decreased stiffness of the back in controls indicates that (for example in pointing) the shoulders rotate further on the axis before hip rotation is required.

#### 3.2.3. Principal Component Analysis with Optimised Feature Parameters

Here, we consider the frequency domain representation of the information to obtain feature vectors using the FFT power spectrum. The PCA based feature orientation along dominant principals axis was used to refine the separation between controls and patients. The Silhouette coefficient was used in the optimisation of feature parameters utilising the result from PCA.

##### Feature Extraction

**Figure 6:**
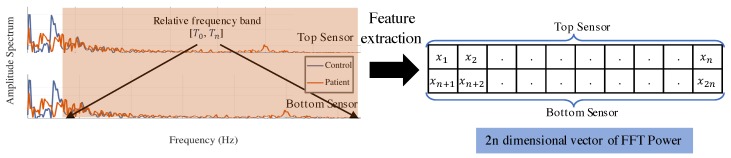
Frequency domain representation of angular rate from the upper sensor and formation of the feature vector with information from the lower sensor.

In each movement routine, angular rate signals $S_{T}$ and $S_{B}$ from upper and lower sensors were considered in the frequency domain [21], where resonance at low frequencies corresponds to the main action, which is periodic. In the relatively higher frequency bands, we expect less information pertaining to normal subject movements. We calculated the spectral power of *n* segments of sub bands to extract a 2*n*-dimensional feature vector from upper and lower sensors. Sub bands are acquired from a cutoff frequency value $T_{0}$ (Hz) to $T_{n}$ (Hz), which is expected to contain particular patient information. Spectral power is calculated as follows in Equation (6):

(6) $$\begin{array}{c} \gamma_{k}=\int_{T_{i}}^{T_{i+1}}Y_{1}{\left(t\right)}^{2}dt,\gamma_{n+k}=\int_{T_{i}}^{T_{i+1}}Y_{2}{\left(t\right)}^{2}dt, \end{array}$$

where k=1,…,n; $\left[T_{i},T_{i+1}\right]$ indicates the relevant frequency band, i=0,…,n−1; $Y_{1}$ and $Y_{2}$ are signals from the upper and lower sensors in the frequency domain. An illustration of feature extraction is presented in Figure 6. Signal power of *n* bands in each sensor formed feature vector $\Gamma_{i}$ for *i*th subject as $\Gamma_{i}=\left[\gamma_{i,1},\gamma_{i,2},\ldots ,\gamma_{i,2n}\right],i=1,\ldots ,m$, where *m* is number of subjects.

In this study, we applied PCA [22,23,24] to our feature dataset of patients and controls to extract principal components from these features. It transformed data into a new coordinate system where the covariance matrix is diagonal. In order to estimate the transformation, we calculated the eigenvectors and eigenvalues of the data covariance matrix. The eigenvector with the largest eigenvalue is the dominant direction of variation. It is considered as the first principal component. The eigenvector with the second largest value is the second principal component and so on. Each direction represented an axis in the new space.

For each movement routine, a set of p-dimensional feature vectors of patients and controls, Ω, is considered as the current space, represented in Equation (7):

(7) $$\Omega ={\left(\begin{array}{cccc} \Gamma_{1} & \Gamma_{2} & \ldots & \Gamma_{m} \end{array}\right)}^{T}.$$

In order to find the principal components in the new space, we used the covariance matrix *K*, which is calculated by Equation (8), to find the eigenvectors $e=\{e_{1},e_{2},\ldots ,e_{p}\}$ and corresponding eigenvalues $\lambda =\{\lambda_{1},\lambda_{2},\ldots ,\lambda_{p}\}$ from Equation (9):

(8) $$K=covar\left(\Omega \right)=\Omega^{T}\Omega ,$$

(9) $$Ke_{i}=\lambda_{i}e_{i},\;$$

where $e_{i}$ is an eigenvector corresponding to eigenvalue $\lambda_{i}$.

The common approach is to rank the eigenvectors from largest to smallest corresponding eigenvalue and choose the top *k* eigenvectors. In our experiment, we selected k=3 to obtain the first three principal components $PC_{1},PC_{2}$, and $PC_{3}$.

To calculate principal components, a weight matrix/transformation matrix $W_{k\times p}$ is formed by selected eigenvectors. The *k*th principal components for the *i*th subject, $P_{i}$, in the new space was computed from the original feature vector $\Gamma_{i}$ as given in Equation (10):

(10) $$P_{i}=\left(\begin{array}{c} PC_{1}\\ PC_{2}\\ \ldots \\ PC_{k} \end{array}\right)=\Gamma_{i}W_{k\times p}^{T}=\left(\begin{array}{cccc} \gamma_{i1} & \gamma_{i2} & \ldots & \gamma_{ip} \end{array}\right)\left(\begin{array}{cccc} e_{11} & e_{21} & \ldots & e_{k1}\\ e_{12} & e_{22} & \ldots & e_{k2}\\ \ldots & \ldots & \ldots & \ldots \\ e_{1p} & e_{k2p} & \ldots & e_{kp} \end{array}\right).$$

Principal components were extracted from the histogram of the power spectrum calculated using Equation (6), which required the parameters of cutoff ($T_{0}$, $T_{n}$) and number of bins (bin_num). We optimised respective parameters using the approach outlined in the next section.

##### Numerical Optimisation of Feature Parameters Using Silhouette Coefficient

**Figure 7:**
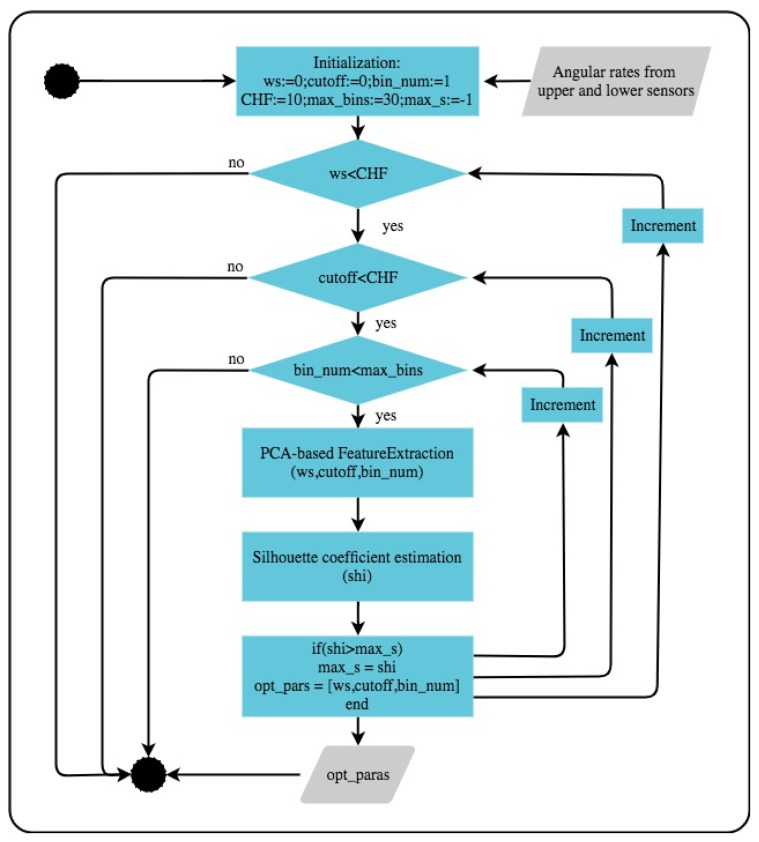
Optimisation for parameters selection.

A numerical optimisation based approach was used to obtain the best parameters that maximise the distinct clustering of patients and controls. Figure 7 describes the procedure used in the underlying numerical optimisation.

The Silhouette coefficient captures the similarity of an object to its own cluster (cohesion) compared to other clusters (separation). The representative numerical value varies from −1 to +1. A higher value for this coefficient (close to +1) indicates that the object is closely associated to its own cluster and well-separated from the neighbouring clusters. A lower value (close to −1) indicates that the object is associated to the alternate cluster. A value of 0 indicates that the object is very close to the decision boundary between the two neighbouring clusters. The average Silhouette value (Ξ) for the overall data set corresponding to a cluster is a measure of how appropriately the data has been clustered. Given two sets of PC features ($P_{i}$) for patients, $A_{1}$ and controls, $A_{2}$, the evaluated value was calculated as in Equations (11)–(14). This measurement was used as a criterion in parameter optimization. In this study, we experimented $\left(ws,bin\_num,T_{0}\right)$ within the specified interval to find the parameter that produced the best clustering based on the silhouette coefficient, i.e., maximizing the coefficient value. Here, $T_{n}=T_{0}+ws$,

(11) $$\alpha \left(P_{i}\right)=\frac{1}{|A_{k}|}\sum_{P_{i},P_{j}\in A_{k},j\neq i}\Delta \left(P_{i},P_{j}\right),$$

(12) $$\beta \left(P_{i}\right)=\min_{\begin{array}{c} P_{i}\in A_{k}\\ l,k\in \{1,2\}\\ l\neq k \end{array}}\frac{1}{|A_{l}|}\sum_{P_{i}\in A_{l}}\Delta \left(P_{i},P_{j}\right),$$

(13) $$\xi_{i}=\frac{\beta_{i}-\alpha_{i}}{\max(\alpha \left(P_{i}\right),\beta \left(P_{i}\right))},i=1,\ldots ,n,$$

(14) $$\Xi =\frac{\sum_{i=1}^{n}\xi_{i}}{|A_{1}\cup A_{2}|},$$

where $\xi_{i}$ indicates silhouette value for the *i*th subject ; *n* is number of subjects (patients and controls); $\alpha (P_{i})$ is the average distance from the *i*th subject to the other subjects in the same cluster as *i* and $\beta (P_{i})$ is the minimum average distance from the *i*th subject to subjects in a different cluster, minimised over clusters; $\Delta (P_{i},P_{j})$ is Euclidean distance between $P_{i}$ and $P_{j}$.

## 4. Experimental Results

**Figure 8:**
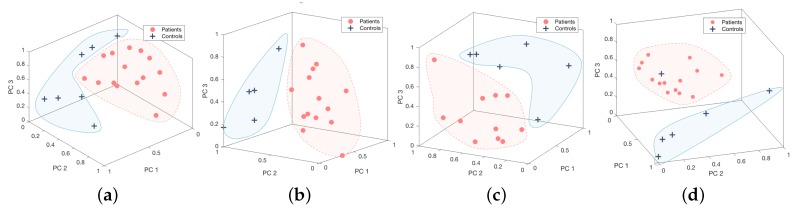
Principal component description of the four movement routines ((**a**) pouring, (**b**) pointing, (**c**) walking, and (**d**) walking around a chair) from Sensor 2 and 3.

**Figure 9:**
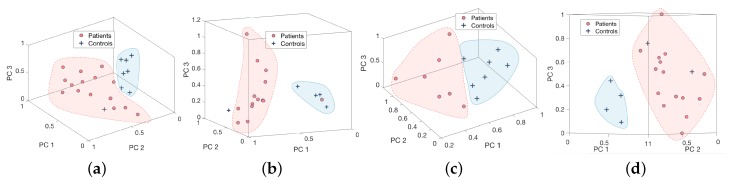
Principal component description of the four movement routines ((**a**) pouring, (**b**) pointing, (**c**) walking, and (**d**) walking around a chair) from Sensor 2 and 4.

Feature vectors are extracted from the higher frequency band and the precise values corresponding to each movement routine are given in s Table 2 and Table 3. It is evident from this study that the relevant frequency band containing the disability related information is generally in the higher frequency range and this was used as the feature for each activity. The dominant frequency band with higher levels of energy corresponds to activity related information and, for the underlying activities, does not contain most of the discriminatory characteristics pertaining to the disability as depicted in Figure 2. Figure 8 demonstrates 3-dimensional features of controls and patients performing four tests from Sensor 2 vs. 3, and Figure 9 for Sensor 2 vs. 4. They are distributed into two clusters where the patients group is in red and controls group is in blue. However, the Silhouette values show that the combination between Sensor 2 vs. 3 is performing significantly better than Sensor 2 vs. 4.

Analysis validation was performed based on the H&Y scale. A linear bivariate correlation, Pearson method, has been applied to find the correspondences between PCs and H&Y score. Table 4 presents high correlation values of 0.78, −0.94, −0.75 for PCs in four movement routines from Sensor 2 vs. 3.

**Figure 10:**
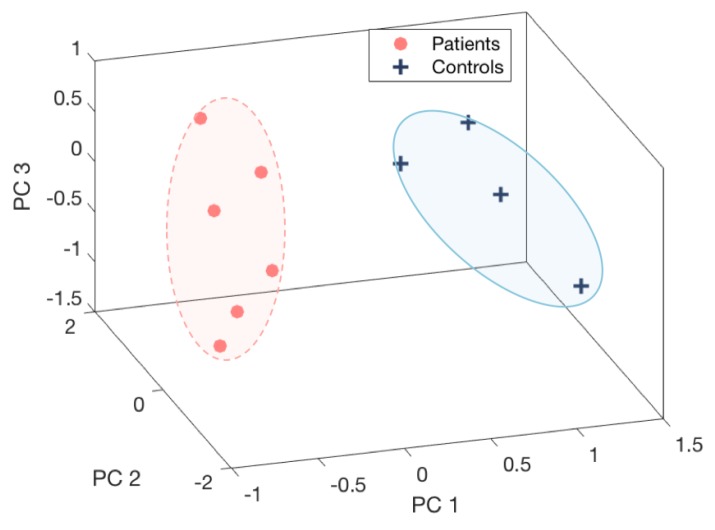
Combination of pouring, pointing, walking, and walking around a chair from Sensor 2 vs. 3.

These PC features of four movement routines were compounded into a combined features vector. Then, we extracted the principal components based on the combined feature vectors. Due to limitation of a clinical nature, not all of subjects were able to performed all four tests. Therefore, the combination of all tests was evaluated based on common subjects including six patients and four controls. The first three PC features of the test combinations were presented in Figure 10. The significant separation of patient and control groups depicted in the figure emphasises the benefit of combining the four movement routines. They also received a high correlation coefficient according to the H&Y score, −0.81 for $PC_{1}$, as depicted in Table 4.

## 5. Conclusions

Physicians typically engage a number of movement routines to identify the bradykinesia, axial rigidity and postural instability of PD. Indeed, gathering information in a clinical environment with expert knowledge is common practice. The patient’s movement information during normal daily activities can provide vital information in the treatment process. Thus, the primary goal of this research was to investigate a number of unstructured movement routines in order to capture PD symptoms to obtain regular information about a patient’s condition when they are in their natural settings. Indeed, the practical sensory requirement was to engage a minimum number of sensors to facilitate the longer term use [4,25]. Furthermore, our objective here is not to completely quantify PD specific changes, which even require the measure of peripheral characteristics [3] as well, but to investigate the possibility of axial rigidity and postural stability captured with minimal sensors during domestic activities. The four movement routines (pouring, pointing, walking, and walking around a chair) we considered, used BioKin${}^{\mathrm{TM}}$ sensors attached to human limbs to capture motion data. The study finds that while same side sensors, pair of Sensor 2 vs. 4, and cross side sensors, pair of Sensor 2 vs. 3, provided significant information, the cross-side combination (Sensor 2 vs. 3) had indeed contained the most information. The information from each sensor pair was analyzed using PCA validated with the level of clustering and correlation with the patient’s medical status represented by the H&Y score. In all four of these unstructured movement routines, the patients could be separated from controls. It was also evident that typically a frequency band in the higher range from the more energy dominant and activity related lower range frequencies contained significant discriminatory information relevant to PD during each activity. This is a preliminary study with a limited number of subjects with clear axial impairments from PD designed to establish whether it was possible to distinguish them from controls in an unstructured laboratory setting. The findings give us confidence to believe that further studies using a broader range of PD subjects and in more naturalistic settings is justified.

## Figures and Tables

**Table 1:** Dataset: number of participants.

Activity	Controls	Patients	Total	H&Y Score
Pouring	8	15	23	1.67 ± 1.6
Pointing	5	14	19	2 ± 1.3
Walking	7	12	19	1.64 ± 1.3
Walking around a chair	6	15	21	1.68 ± 1.4

**Table 2:** Optimised parameters for Sensor 2 vs. 3 combination.

Activity	Number of Bins	Cutoff $\left(T_{0}\right)$	Window Size (ws)	Frequency Band $\left(T_{0},T_{n}\right)$	Silhouette Coefficient
Pouring	19	4.5	0.5	[4.5–5]	0.27 > 0
Pointing	10	6.4	1.2	[6.4–7.6]	0.47 > 0
Walking	9	3.5	1.5	[3.5–5]	0.34 > 0
Walking around a chair	3	0.2	2.4	[0.2–2.6]	0.34 > 0

**Table 3:** Optimised parameters for Sensor 2 vs. 4 combination.

Activity	Number of Bins	Cutoff $\left(T_{0}\right)$	Window Size (ws)	Frequency Band $\left(T_{0},T_{n}\right)$	Silhouette Coefficient
Pouring	24	2.25	7	[2.25–9.25]	0.33 > 0
Pointing	23	0.6	2.6	[0.6–3.2]	0.43 > 0
Walking	16	4.25	5	[4.25–9.25]	0.32 > 0
Walking around a chair	11	0.2	2.2	[0.2–2.4]	0.31 > 0

**Table 4:** Correlation between analysis results from four tests combination and H&Y score from Sensor 2 vs. 3.

Activity	Pearson Correlation		
	*PC*_1_	*PC*_2_	*PC*_3_
Pouring	−0.21	0.78	0.002
Pointing	0.62	−0.94	−0.13
Walking	−0.56	0.73	−0.27
Walking around a chair	−0.75	0.61	0.93
Combination of all tests	−0.81	−0.16	0.52
